# Cardioprotective effectiveness of SGLT2 inhibitors in older diabetic women with early-stage breast cancer following anthracycline- and/or trastuzumab-based treatment

**DOI:** 10.1177/17588359251378245

**Published:** 2025-09-23

**Authors:** Yi-Shao Liu, Jamie C. Barner, Kenneth A. Lawson, Yan Liu, Chanhyun Park

**Affiliations:** Health Outcomes Division, College of Pharmacy, The University of Texas at Austin, Austin, TX, USA; Health Outcomes Division, College of Pharmacy, The University of Texas at Austin, Austin, TX, USA; Health Outcomes Division, College of Pharmacy, The University of Texas at Austin, Austin, TX, USA; Department of Internal Medicine, Dell Medical School, The University of Texas at Austin, Austin, TX, USA; Health Outcomes Division, College of Pharmacy, The University of Texas at Austin, 2409 University Avenue, Austin, TX 78712, USA

**Keywords:** anthracyclines, cardio-oncology, cardiotoxicity, cardiovascular diseases, SGLT2 inhibitors, trastuzumab

## Abstract

**Background:**

Real-world evidence on protective effects of sodium-glucose cotransporter-2 inhibitors (SGLT2i) against anthracycline- or trastuzumab-induced cardiotoxicity in patients with breast cancer is limited.

**Objectives:**

To examine the cardioprotective benefits of SGLT2i in older women with early-stage breast cancer (EBC) following anthracycline- and/or trastuzumab-based therapies.

**Design:**

This was a retrospective cohort study using the 2011–2019 SEER-Medicare database.

**Methods:**

We identified women aged over 65.5 years and diagnosed with stage I–III BC who received anthracycline and/or trastuzumab and subsequently initiated antidiabetic medications. Propensity scores were used to match one new-user episode of SGLT2i with four new-user episodes of other antidiabetic medications (OAMs). The primary outcome was a composite endpoint consisting of heart failure (HF), stroke, myocardial infarction, and arrhythmia. Secondary outcomes included hospitalization due to HF (HHF) and incident HF or cardiomyopathy (CM). Cause-specific hazard ratios (csHR) between SGLT2i and OAMs groups were assessed for each outcome, with all-cause death treated as a competing event.

**Results:**

From 1195 women examined, 1777 new-user episodes were identified. After 1:4 matching, there were 131 episodes in the SGLT2i group and 469 in the OAM group. Covariates were well-balanced between groups. No statistically significant differences were observed in the composite cardiovascular (csHR = 0.71; 95% confidence interval (CI): 0.44–1.15; *p* = 0.24), HHF (csHR = 0.92; 95% CI: 0.10–8.27; *p* = 0.94), or incident HF/CM (csHR = 0.77; 95% CI: 0.45–1.34; *p* = 0.36) outcomes. Results were consistent across individual SGLT2i and clinical subgroups, including those with/without established cardiovascular diseases and those exposed to various cardiotoxic cancer treatments.

**Conclusion:**

No significant differences in cardiovascular risks were found between women with EBC who initiated SGLT2i versus OAMs after anthracycline or trastuzumab treatments, which might be due to the limited sample size. Further investigation through clinical trials is necessary to confirm the cardioprotective potential of SGLT2i among patients with EBC.

## Plain language summary

Heart protection benefits of SGLT2 inhibitors in early breast cancer after chemotherapy

Breast cancer treatments such as anthracyclines and trastuzumab can increase the risk of heart problems. Some studies suggest that a class of diabetes medications called sodium-glucose cotransporter-2 inhibitors (SGLT2i) may help protect the heart. However, real-world data on their effectiveness in breast cancer patients is limited. We examined whether SGLT2i can reduce heart-related complications in older women with early-stage breast cancer who received anthracycline- or trastuzumab-based therapies. Using Medicare data from 2011 to 2019, we identified women over the age of 65.5 who started taking diabetes medications after cancer treatment. We compared those who took SGLT2i with those who took other antidiabetic medications (OAMs), ensuring the groups were similar using a statistical method called propensity score matching. Our study focused on heart-related outcomes, including heart failure, stroke, heart attacks, and irregular heartbeats. We also looked at hospitalizations due to heart failure and new cases of heart failure or weakened heart function. Among the 1,195 women analyzed in our study, 131 episodes of SGLT2i use were compared with 469 episodes of OAM use. The results showed no significant differences between the two groups in terms of heart-related problems. This means that women who took SGLT2i did not appear to have a lower risk of heart complications compared to those who took other diabetes medications. Although the findings suggest no clear heart benefits of SGLT2i in this population, the small sample size may have affected the results. More research, especially clinical trials, is needed to better understand whether SGLT2i can help protect the hearts of patients with breast cancer.

## Introduction

Antineoplastic agents play a crucial role in reducing breast cancer mortality, but they also have negative side effects, notably cardiotoxicity.^1,2^ Some standard treatments for early-stage breast cancer (EBC), such as anthracycline-based chemotherapies^3^ and targeted therapy (e.g., trastuzumab)^4,5^ carry a significant risk of cardiotoxicity, further resulting in the occurrence of heart failure (HF). These cardiovascular complications are especially critical in older adults with cancer, who often have a higher burden of baseline cardiovascular diseases (CVDs) and comorbid conditions.^6^ Indeed, CVDs are becoming the second leading cause of death in older women with EBC.^7,8^ Consequently, this has spurred an increase in research focusing on drugs with cardioprotective potential.^9–18^

Sodium-glucose cotransporter-2 inhibitors (SGLT2i) have recently gained scientific interest due to their well-established cardioprotective profile among patients with type 2 diabetes (T2DM).^19,20^ Several possible mechanisms were established to explain the cardioprotective effects of SGLT2i. These underlying mechanisms include metabolism alteration, improvements in ventricular loading conditions, inhibitions of electrolyte exchange and adipokines, and prevention of cardiac fibrosis.^20^ Beyond this, preliminary data from in vitro studies and animal models suggest that SGLT2i may mitigate cardiotoxicity caused by anthracyclines or trastuzumab.^21^ Despite these promising results, research comparing the cardiac outcomes between SGLT2i versus other antidiabetic medications (OAMs) in diabetic patients with cancer who received anthracycline or trastuzumab is limited.

Therefore, our study aims to address this knowledge gap by using a new-user episode-based design to evaluate the potential cardioprotective benefits of SGLT2i in older women with EBC following anthracycline- and/or trastuzumab-based therapies.

## Methods

### Data source

In this retrospective study, we used the Surveillance, Epidemiology, and End Results (SEER)-Medicare dataset, which combines SEER registry data with Medicare enrollment and claims data. Approximately 95% of individuals aged 65 and older in the SEER files are linked to the Medicare enrollment file. The SEER program, supported by the National Cancer Institute, gathers information on individuals who were newly diagnosed with cancer from multiple state registries, covering approximately 26.5% of the US population.^22^ The SEER data includes demographic characteristics and breast cancer-specific information. The Medicare data provides information on health service utilization of insured beneficiaries.^23^ The study protocol was reviewed and approved by the Institutional Review Board (IRB) of the University of Texas at Austin. This study follows the STROBE cohort reporting guidelines (Supplemental File).

### Study sample and design

**Figure 1.**
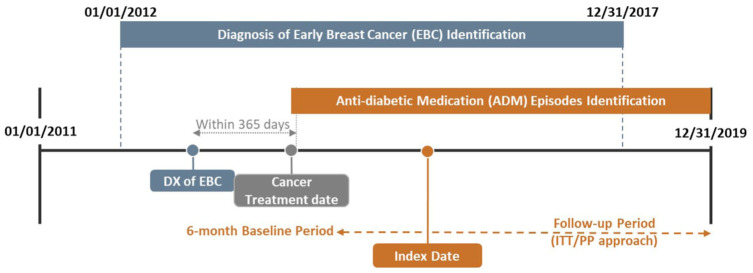
Study design overview. DX, diagnosis; ITT, intention-to-treat; PP, per-protocol.

Women aged ⩾65.5 years with localized or regional breast cancer who received anthracyclines- or trastuzumab-containing therapies within 365 days of their first diagnosis of breast cancer between January 1, 2012, and December 31, 2017, were identified from the SEER-Medicare data.^24^ The age threshold of 65.5 years was chosen to align with the structure of the SEER-Medicare data, which primarily includes individuals aged 65 and older, and to ensure a 6-month pre-index period for assessing baseline covariates. The cancer treatment date was defined as the first date of anthracyclines or trastuzumab initiation. Furthermore, we identified the episodes of newly initiated SGLT2i or OAM any time after the cancer treatment date among these older women. New-user episodes were identified when there were no prior prescriptions for the same class of antidiabetic medication within the preceding 6 months. The index date referred to the date on which the first prescription from the selected antidiabetic medication classes was issued or filled between January 1, 2013, and December 31, 2019. As a result, a single patient could contribute several new-user episodes (Figure 1).

Additional episode-based inclusion criteria were as follows: (1) women must be 65.5 years or older on the index date; (2) women must have been diagnosed with T2DM on or before the index date; (3) a minimum of 30 consecutive days of the same-class medication supply was used as a proxy indicating that a women had been consistently taking the antidiabetic medication for at least 1 month; and (4) they must have maintained continuous enrollment in Medicare Part A, Part B, and Part D coverage for a minimum of 6 months before the index date (referred to as the pre-index period) up to the last available tracking date in the dataset. Episodes were excluded if: (1) they had any cancer diagnoses before the primary diagnosis of EBC; (2) they were enrolled in a health management organization at any time during the 6-month pre-index period or from the index date to the last available tracking date; (3) they were enrolled in Medicare due to end-stage renal disease or disability; or (4) they received a diagnosis of type 1 diabetes before the index date. Episodes with SGLT2i initiation were classified as the SGLT2i group, while the control group consisted of episodes where OAM was initiated.

### Study endpoints and follow-up approaches

The primary outcome of this study was a composite endpoint that encompassed the diagnosis of HF, stroke, myocardial infarction (MI), and arrhythmia. The primary outcome was identified using the International Classification of Diseases, Ninth (ICD-9) and Tenth Revision (ICD-10) diagnosis codes from various Medicare files (Supplemental Table 1). Secondary outcomes included: (1) hospitalizations due to heart failure (HHF), which were identified using ICD-9/ICD-10 diagnosis codes from the inpatient file of Medicare, with HF as the primary diagnosis and (2) a composite endpoint consisting of a diagnosis of HF and cardiomyopathy (CM) identified from all included Medicare files with ICD-9/ICD-10 diagnosis information (Supplemental Table 1). The incidence rate of events was generated by dividing the count of events by the cumulative person-years at risk. This was represented as events per 100 person-years with 95% confidence intervals (CI).

Women fulfilling eligibility criteria were followed up from the index date until the outcome occurrence, while all-cause death was treated as a competing risk. In the intention-to-treat (ITT) analysis, patients were censored either if they altered their Medicare A, B, or D enrollment status or reached the end of the study period (December 31, 2019). To assess the study’s robustness, a per-protocol (PP) follow-up approach was used for the sensitivity analysis. Under this framework, each episode was additionally followed from the initiation of the index treatment until the end of the days’ supply plus a grace period. This grace period was defined as the number of days’ supply of the last observed prescription for that specific medication class.

### Statistical analysis

This study reported continuous variables as mean values with associated standard deviations (SDs), while categorical variables were represented as frequencies and percentages. Descriptive statistics for all the baseline characteristics were reported both before and after propensity score matching (PSM), comparing the OAM and SGLT2i groups. The propensity score for SGLT2i initiation was determined using a non-parsimonious multivariable logistic regression model conditional on all covariates listed in Supplemental Table 2.^25^ Covariates included in the propensity score model were selected based on clinical relevance, published literature, and input from oncology and cardiology clinicians to ensure adequate adjustment for potential confounding. A 1:4 modified greedy nearest neighbor matching within a caliper width of 0.2 times the SD of the logit of the propensity score was performed without replacement.^26^ The adequacy of covariate balance between groups after matching was evaluated using pooled standardized mean difference (SMD), with a value of less than 0.25 indicating reasonable balance between groups.^27,28^

To assess the risk of primary and secondary outcomes over time in the SGLT2i and OAM groups, we utilized cumulative incidence functions (CIF), treating all-cause death as a competing risk.^29^ The statistical significance of differences in CIF between the groups was assessed using Fine-Gray subdistribution hazard models. Moreover, univariable cause-specific hazard models with all-cause death as a competing risk event were used to compare the risk of time-to-first events between SGLT2i and OAM groups among the women who remained alive.^29^ The cause-specific hazard ratios (csHR) with estimated 95% CIs were presented for each outcome. Considering the presence of our cluster-correlated data, where a single patient may have multiple treatment initiation episodes, a robust variance estimator was applied within these models.^30,31^ This method adjusts standard errors to account for within-patient clustering and is commonly used in survival analyses involving repeated measures.^32^ By clustering on patient ID, the robust variance estimator accounts for intra-individual correlation, enabling valid statistical inference despite non-independent observations.

Analyses for individual outcomes, including PSM and time-to-event analyses, were repeated across multiple prespecified subgroups, including individual SGLT2i, presence or absence of previous CVDs, and cardiotoxic cancer treatments (anthracycline-only and trastuzumab). Given the potentially limited sample sizes within these subgroups, these analyses were considered exploratory and intended to generate hypotheses rather than support definitive conclusions. All statistical analyses were conducted using SAS Version 9.4 (SAS Institute Inc., Cary, NC, USA). A significance level (α) was set at 0.05 for all analyses of two-tailed tests.

## Results

### Patient characteristics

**Figure 2.**
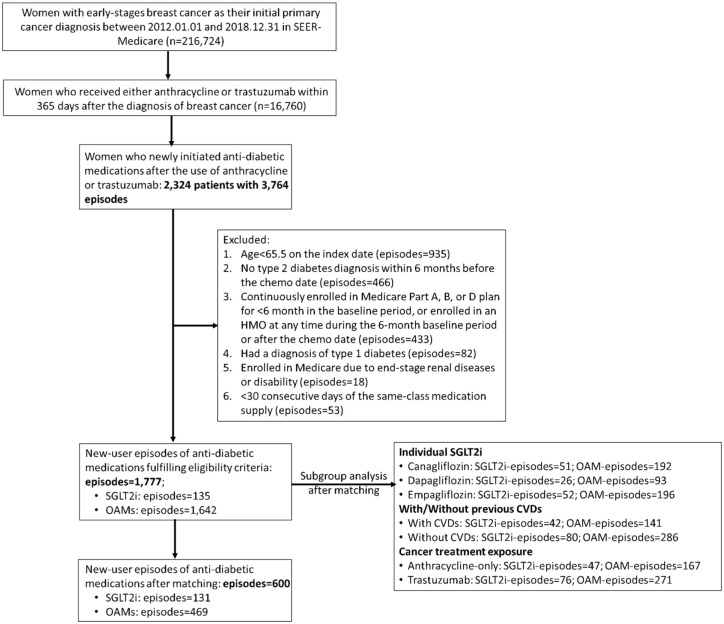
Patient flow diagram. CVDs, cardiovascular diseases; OAM, other antidiabetic medication; SEER, the Surveillance, Epidemiology, and End Results; SGLT2i, sodium-glucose cotransporter-2 inhibitors.

A total of 3764 new-user episodes were identified from 2364 women who newly initiated antidiabetic medications after the use of anthracycline and/or trastuzumab. Of these, we identified 1777 new-user episodes of antidiabetic medications fulfilling eligibility criteria; 135 (7.6%) episodes were in the SGLT2i group, while 1642 (92.4%) episodes were in the OAM group. After PSM, we included 131 SGLT2i episodes and 469 OAM episodes from 437 women (Figure 2).

Before 1:4 PSM, the mean age of the 135 episodes receiving SGLT2i was 71.5 (±4.1) years, compared to 72.6 (±5.1) years for those in the OAM group. As compared to the OAM cohort, Table 1 shows the SGLT2i group had a lower prevalence of MI, angina, and warfarin usage, but had a higher prevalence of using metformin, sulfonylureas, thiazolidinediones (TZD), dipeptidyl peptidase 4 inhibitors (DPP4i), and glucagon-like peptide 1 agonists (GLP1a). After 1:4 PSM, the two groups achieved a balanced distribution of measured baseline characteristics (Table 1).

**Table 1.** Baseline characteristics of new-user episodes in SGLT2i and OAM groups before and after 1:4 propensity score matching.

*N* ^a^	Pre-matching			Post-matching		
	SGLT2i	OAM	SMD	SGLT2i	OAM	SMD
	135	1642		131	469	
Sociodemographics						
Mean age, years	71.5 (4.1)	72.6 (5.1)	0.23	71.6 (4.0)	71.7 (4.1)	<0.01
Race/Ethnicity			0.06			0.05
White	91 (67.4)	1135 (69.1)		89 (67.9)	313 (66.7)	
Black	22 (16.3)	255 (15.5)		21 (16.0)	74 (15.8)	
Asian/Hispanics^b^	⩾11	173 (10.5)		⩾11	63 (13.4)	
Others^b,c^	<11	79 (4.8)		<11	19 (4.0)	
Region			0.15			0.08
Northeast	47 (34.8)	587 (35.8)		46 (35.1)	157 (33.5)	
Midwest^b^	⩾11	124 (7.6)		<11	45 (9.6)	
South	32 (23.7)	367 (22.4)		31 (23.7)	110 (23.5)	
West	45 (33.3)	548 (33.4)		44 (33.6)	157 (33.5)	
Others^b^	<11	16 (1.0)		<11	0 (0.0)	
Metro area	116 (85.9)	1404 (85.5)	0.01	112 (85.5)	398 (84.9)	0.02
Marital status			0.07			0.02
Single	16 (11.9)	166 (10.1)		16 (12.2)	55 (11.7)	
Married	45 (33.3)	574 (35.0)		44 (33.6)	157 (33.5)	
Others	74 (54.8)	902 (54.9)		71 (54.2)	257 (54.8)	
Poverty			0.10			0.07
<10% poverty	45 (33.3)	613 (37.3)		43 (32.8)	161 (34.3)	
10%–20% poverty	41 (30.4)	455 (27.7)		40 (30.5)	150 (32.0)	
⩾20% poverty	⩾38	448 (27.3)		⩾37	129 (27.5)	
Unknown^b^	<11	126 (7.7)		<11	29 (6.2)	
Comorbidities						
Heart failure	11 (8.2)	237 (14.4)	0.20	11 (8.4)	37 (7.9)	0.02
Cardiomyopathy^b^	<11	96 (5.9)	0.02	<11	29 (6.2)	0.04
Stroke^b^	<11	102 (6.2)	0.12	<11	17 (3.6)	0.01
MI^b^	<11	81 (4.9)	0.25	<11	<11	0.01
Angina^b^	<11	85 (5.2)	0.26	<11	<11	0.04
Peripheral vascular diseases	18 (13.3)	267 (16.3)	0.08	18 (13.7)	57 (12.2)	0.05
Atrial fibrillation^b^	<11	200 (12.2)	0.22	<11	28 (6.0)	<0.01
Valvular heart diseases	18 (13.3)	234 (14.3)	0.03	18 (13.7)	56 (12.0)	0.05
Arrhythmia^b^	<11	25 (1.5)	0.18	<11	<11	0
Hyperlipidemia	105 (77.8)	1289 (78.5)	0.02	101 (77.1)	376 (80.2)	0.07
Hypertension	118 (87.4)	1475 (89.8)	0.08	115 (87.8)	421 (89.8)	0.06
Chronic kidney diseases	18 (13.3)	349 (21.3)	0.21	18 (13.7)	75 (16.0)	0.06
Hypoglycemia^b^	<11	42 (2.6)	0.02	<11	12 (2.6)	0.02
COPD	16 (11.9)	165 (10.1)	0.06	16 (12.2)	48 (10.2)	0.06
Obesity	38 (28.2)	429 (26.1)	0.05	37 (28.2)	126 (26.7)	0.04
Co-medications						
Dexrazoxane^b^	<11	34 (2.1)	0.11	<11	<11	0.03
Aspirin^b^	<11	28 (1.7)	0.02	<11	<11	0.01
Beta-blocker	59 (43.7)	748 (45.6)	0.04	59 (45.0)	202 (43.1)	0.04
ACEI	54 (40.0)	550 (33.5)	0.14	51 (38.9)	177 (37.7)	0.02
ARBs	50 (37.0)	534 (32.5)	0.09	49 (37.4)	171 (36.5)	0.02
Statin	97 (71.9)	1052 (64.1)	0.17	93 (71.0)	331 (70.6)	<0.01
Calcium-channel blockers	40 (29.6)	472 (28.8)	0.02	39 (29.8)	140 (29.9)	<0.01
Diuretics	50 (37.0)	704 (42.9)	0.12	48 (36.6)	174 (37.1)	<0.01
DOACs^b^	<11	144 (6.9)	0.01	<11	28 (6.0)	0.04
P2y12 inhibitors^b^	<11	113 (6.9)	0.07	<11	20 (4.3)	0.05
Warfarin^b^	<11	60 (3.7)	0.28	<11	<11	<0.01
Insulin	33 (24.4)	254 (15.5)	0.23	33 (25.2)	112 (23.9)	0.03
Metformin	101 (74.8)	683 (41.6)	0.72	97 (74.1)	341 (72.7)	0.03
Sulfonylureas	54 (40.0)	459 (28.0)	0.26	51 (38.9)	165 (35.2)	0.07
Thiazolidinediones	16 (11.9)	71 (4.3)	0.28	14 (10.7)	33 (7.0)	0.12
DPP4i	52 (38.5)	260 (15.8)	0.53	48 (36.6)	150 (32.0)	0.10
GLP1a	19 (14.1)	63 (3.8)	0.36	17 (13.0)	43 (9.2)	0.12
Cancer-related factors						
Breast-conserving surgery	77 (57.0)	931 (56.7)	0.01	74 (56.5)	265 (56.5)	<0.01
Mastectomy	58 (43.0)	823 (50.1)	0.14	57 (43.5)	212 (45.2)	0.03
SEER summary stage			0.22			0.04
Localized	63 (46.7)	701 (42.7)		62 (47.3)	218 (46.5)	
Regional	72 (53.3)	941 (57.3)		69 (52.7)	251 (53.5)	
Grade			0.26			0.08
Well-differentiated	15 (11.1)	99 (6.0)		15 (11.5)	41 (8.7)	
Moderately differentiated	37 (27.4)	559 (34.0)		36 (27.5)	132 (28.1)	
Poorly-differentiated	77 (57.0)	905 (55.1)		74 (56.5)	273 (58.2)	
Undifferentiated^b^	<11	<11		<11	<11	
Not stated^b^	<11	70 (4.3)		<11	23 (4.9)	
Subtype			0.27			0.10
HR+/HER2−	29 (21.5)	534 (32.5)		29 (22.1)	116 (24.7)	
HER2+	77 (57.0)	812 (49.5)		74 (56.5)	264 (56.3)	
TNBC^b^	⩾11	220 (13.4)		⩾11	63 (13.4)	
Unknown^b^	<11	76 (4.6)		<11	26 (5.5)	
Size			0.23			0.11
<2 cm	45 (33.3)	432 (26.3)		43 (32.8)	151 (32.2)	
2–5 cm	44 (32.6)	628 (38.3)		43 (32.8)	169 (36.0)	
>5 cm	16 (11.9)	125 (7.6)		16 (12.2)	44 (9.4)	
Unknown	30 (22.2)	457 (27.8)		29 (21.6)	105 (22.4)	
Radiation	52 (38.5)	618 (37.6)	0.02	51 (38.9)	177 (37.7)	0.02
Chemotherapy			0.15			0.04
Anthracycline-only	52 (38.5)	748 (45.6)		51 (38.9)	188 (40.0)	
Trastuzumab	83 (61.5)	894 (54.4)		80 (61.1)	281 (60.0)	
Other factors						
Index year			0.51			0.16
2012^b^	<11	20 (1.2)		NA	NA	
2013^b^	<11	59 (3.6)		NA	NA	
2014^b^	<11	133 (8.1)		<11	13 (2.8)	
2015	11 (8.2)	200 (12.2)		⩾11	51 (10.8)	
2016	19 (14.1)	268 (16.3)		19 (14.5)	54 (11.5)	
2017	36 (26.7)	302 (18.4)		34 (26.0)	121 (25.8)	
2018	24 (17.8)	347 (21.1)		23 (17.6)	97 (20.7)	
2019	41 (30.4)	313 (19.1)		40 (30.5)	133 (28.4)	
Time gap			0.45			0.08
⩽180 days, *n* (%)^b^	<11	336 (20.5)		<11	43 (9.2)	
>180 and ⩽365 days, *n* (%)^b^	⩾11	243 (14.8)		⩾11	53 (11.3)	
>365 days, *n* (%)	112 (83.0)	1063 (64.7)		109 (83.2)	373 (79.5)	

a Continuous variables are presented as mean (±SD) while categorical variables are presented as n (%).

b Numbers less than 11 are suppressed per SEER-Medicare data use agreement.

c Including Asian, Hispanics, American Indians and Alaskan Natives, and unknown

ACEIs, angiotensin-converting enzyme inhibitors; ARBs, angiotensin receptor blockers; COPD, chronic obstructive pulmonary disease; DOACs, direct oral anticoagulants; DPP4i, dipeptidyl peptidase 4 inhibitor; GLP-1a, glucagon-like peptide-1 agonists; HER2, human epidermal growth factor 2 receptor; HR, hormone receptor; OAM, other antidiabetic medication; SD, standard deviation; SEER, the Surveillance, Epidemiology, and End Results; SGLT2is, sodium-glucose cotransporter-2 inhibitors; SMD, standardized mean difference; TNBC, triple-negative breast cancer.

### Clinical outcomes

In the ITT analysis, the median follow-up period for the cardiovascular composite endpoint was 1.39 years for episodes in the SGLT2i group and 1.36 years for those in the OAM group. The crude incidence rate for the cardiovascular composite endpoint was 6.9 per 100 person-years (95% CI = 4.2–11.1) in the SGLT2i group, versus 9.8 per 100 person-years (95% CI = 7.9–12.1) in the OAM group. Regarding HHF, the rate was 0.4 per 100 person-years (95% CI = 0.0–3.0) in the SGLT2i group and 0.5 per 100 person-years (95% CI = 0.0–3.0) in the comparison group. The incidence rate for new HF or CM diagnoses was 5.4 per 100 person-years (95% CI = 3.1–9.4) among those initiating SGLT2i, compared to 7.1 per 100 person-years (95% CI = 5.5–9.1) among those starting on OAM.

**Figure 3.**
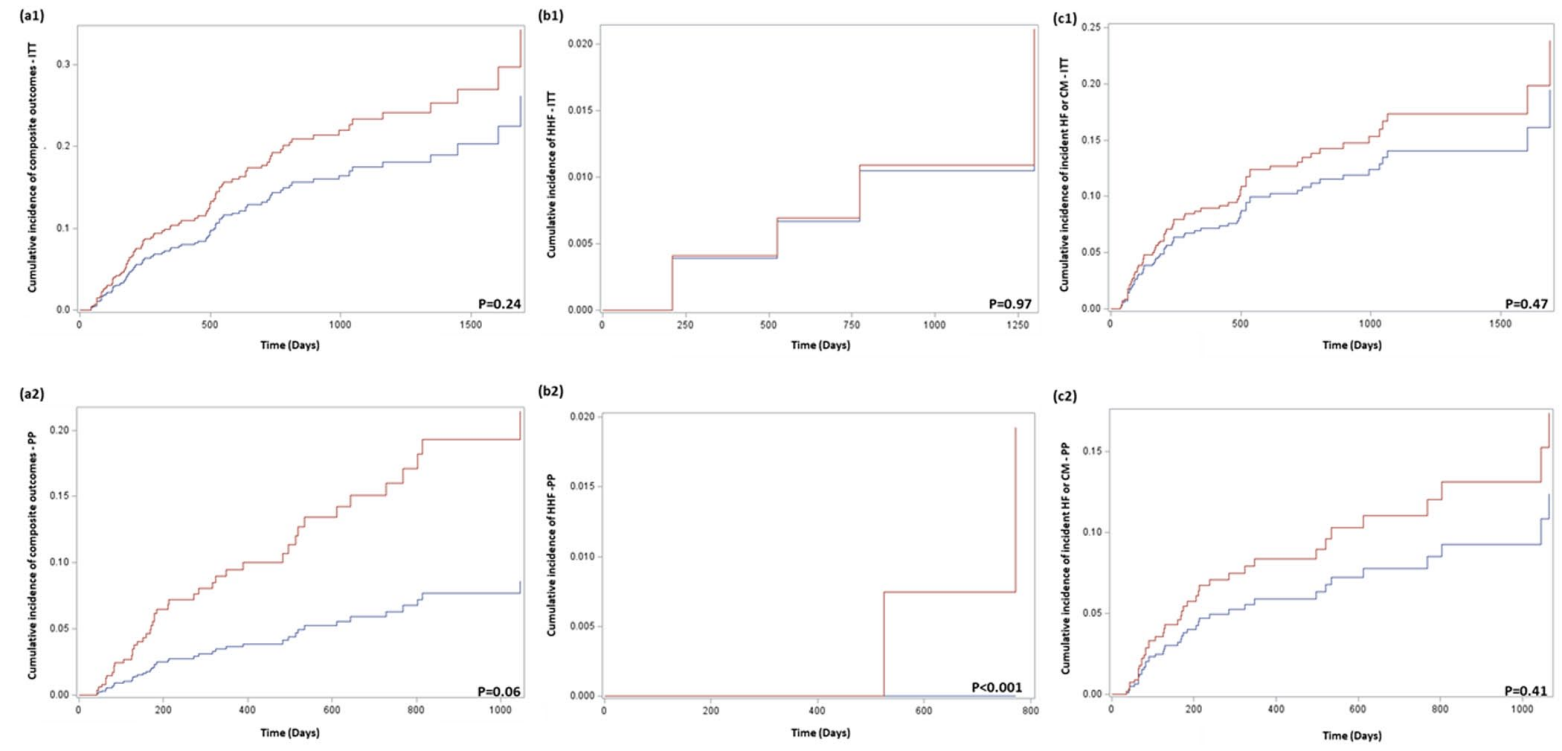
CIF curves across primary and secondary outcomes based on ITT and PP follow-up approaches. CIF curves of (a) cardiovascular composite outcomes, (b) hospitalization due to heart failure, and (c) incident heart failure or cardiomyopathy diagnoses using (1) in intention-to-treat and (2) per-protocol follow-up approaches. The *p*-values are obtained from Fine-Gray subdistribution hazard models after 1:4 propensity score matching. Curves are shown in red for OAM-initiated episodes and blue for SGLT2i-initiated episodes. ITT, intention-to-treat; OAM, other antidiabetic medication; PP, per-protocol; SGLT2i, sodium-glucose cotransporter-2 inhibitors.

Figure 3 shows the CIF curves of the cardiovascular composite outcome, HHF, and incident HF or CM stratified by SGLT2i and OAM initiation among women receiving either anthracycline or trastuzumab in both ITT and PP analyses. This figure indicates that the estimated cumulative risks for the majority of cardiovascular outcomes had no statistical significance between the SGLT2i and OAM groups, regardless of the follow-up approaches; however, the zero HHF event identified from the SGLT2i group using the PP approach resulted in a significance at *p* < 0.001.

The csHR of the cardiovascular composite endpoint associated with SGLT2i initiation was 0.71 (95% CI = 0.44–1.15; *p* = 0.19), indicating no significant difference in risk between the SGLT2i and OAM groups. The analysis of HHF risk revealed no significant difference, with a csHR of 0.92 (95% CI = 0.43–1.98; *p* = 0.94) associated with the SGLT2i group. Regarding incident HF or CM diagnoses after anthracycline or trastuzumab use, the csHR for SGLT2i initiation was 0.77 (95% CI = 0.45–1.34; *p* = 0.36) as compared to OAM initiation, also showing no significant difference between the cohorts. Consistent with findings from the ITT analysis, a nonsignificant trend toward a reduced risk estimate was observed for the composite CVD and new HF or CM diagnosis. However, zero events of HHF in the SGLT2i group resulted in a csHR of 0 (*p* < 0.001). Table 2 summarizes the crude incidence rates and csHR for both the ITT and PP analyses.

**Table 2.** Event counts, crude incidence rates, and hazard ratios for episodes initiated with SGLT2i or OAM using intention-to-treat and per-protocol follow-up approaches.

Outcomes	Intention-to-treat analysis		Per-protocol analysis	
	OAM	SGLT2i	OAM	SGLT2i
*N*	469	131	469	131
Cardiovascular composite endpoint (HF, stroke, MI, and arrhythmia)				
Event counts (crude incidence rate per 100 person-years)	76 (9.8)	15 (6.9)	41 (9.0)	<11 (3.3)
csHR (95% CI)	1.0 (Ref)	0.71 (0.44–1.15), *p* = 0.19	1.0 (Ref)	0.37 (0.13–1.00), *p* = 0.05
HHF				
Event counts^a^ (crude incidence rate per 100 person-years)	<11 (0.5)	<11 (0.4)	<11 (0.4)	0 (0.0)
csHR (95% CI)	1.0 (Ref)	0.92 (0.43–1.98), *p* = 0.94	1.0 (Ref)	0, *p* < 0.001
Incident HF or CM				
Event counts (crude incidence rate per 100 person-years)	57 (7.1)	12 (5.4)	34 (7.5)	<11 (5.0)
csHR (95% CI)	1.0 (ref)	0.77 (0.45–1.34), *p* = 0.36	1.0 (Ref)	0.67 (0.30–1.49), *p* = 0.33

a Numbers less than 11 are suppressed per SEER-Medicare data use agreement.

CI, confidence intervals; CM, cardiomyopathy; csHR, cause-specific hazard ratio; HF, heart failure; HHF, hospitalization due to heart failure; MI, myocardial infarction; OAM, other antidiabetic medications; SGLT2i, sodium-glucose cotransporter-2 inhibitors.

### Subgroup analysis

Results across all subgroups (three individual SGLT2i, presence and absence of previous CVDs, and anthracyclines-only or trastuzumab cancer treatment exposure) were consistent with the main analyses. Although there was a tendency toward lower point estimates for the cardiovascular composite endpoint and incident HF or CM in the SGLT2i group compared to the OAM group, the differences in the risk for all CV outcomes did not reach statistical significance (Supplemental Tables 3 and 4).

## Discussion

Our study used a new-user episode-based design and the SEER-Medicare dataset to mitigate confounding effects in real-world evidence studies.^33,34^ Among older women with T2DM and EBC who received anthracyclines or trastuzumab, we found no statistically significant differences in cardiovascular risks between those who newly initiated SGLT2i and those who newly used OAMs. This lack of significant differences was consistent across both ITT and PP follow-up approaches, as well as across multiple patient subgroups. Notably, although the differences were not statistically significant, consistently lower point estimates were observed in all analytical approaches and prespecified subgroups, suggesting a potential trend toward cardioprotective benefits with SGLT2i use.

In a population-based study in Canada, Abdel-Qadir et al. examined 933 patients with cancer receiving anthracycline-containing therapy in the Canadian Ontario Health Insurance Plan (*N* = 933, 2016–2019), revealing that SGLT2i use did not lead to a statistically significant reduction in the risk of incident HF (csHR = 0.55, 95% CI = 0.23–1.31; *p* = 0.18) or any CVD diagnosis (csHR = 0.39, 95% CI = 0.12–1.28; *p* = 0.12) when compared with non-SGLT2i users.^35^ These nonsignificant findings were consistent with our results, but our study extends the work of Abdel-Qadir et al. by incorporating comprehensive cancer-related covariates and considering both anthracycline and trastuzumab users. In addition, a new-user episode-based design was adopted in our study to reduce the bias caused by mixing new users with existing SGLT2i users. By including active comparators of SGLT2is, our study could greatly reduce confounding by indication and other unmeasured baseline covariates.^33,34^

A population-based study using TriNetX research network database (*N* = 1,280, 2013–2020) by Avula et al. found that adding SGLT2i to contemporary guideline-directed medical therapy significantly reduced HF exacerbations (HR = 0.62, 95% CI = 0.47–0.81; *p* < 0.001) and atrial fibrillation/flutter (HR = 0.52, 95% CI = 0.28–0.95; *p* = 0.03) compared to non-SGLT2i users.^36^ Another study using the same database also reported reductions in HF exacerbations and cancer therapy-related cardiac dysfunction (CTRCD).^37^ However, these significant results may be due to a longer follow-up period and better detection of asymptomatic Left Ventricular Ejection Fraction (LVEF) reductions. Our new-user episode-based design with active comparators might attenuate differences between SGLT2is and OAMs, leading to more precise but potentially null results.

Although neither the ITT nor the PP approaches in our study showed statistically significant results, the PP analysis consistently yielded lower point estimates compared to the ITT analysis across all outcomes of interest. ITT tends to attenuate the difference between treatment groups due to therapy discontinuation or treatment crossover. Multiple RCTs that have adopted both methods reported a more pronounced difference in hazard ratios between treatment arms when the PP approach was applied.^38^ The lower csHR of the SGLT2i group in our study, based on the PP approach, may lead to the hypothesis that ongoing treatment with SGLT2is could translate into reducing risks of adverse cardiovascular outcomes. However, it remains unclear whether this effect is due to adherence or the underlying characteristics of patients who were more likely to comply.^38^ Therefore, we implemented both ITT and PP analyses to provide a comprehensive view of the cardioprotective benefits of SGLT2i versus OAM in managing cardiotoxic cancer therapies.

The statistically insignificant results across all outcomes of interest between SGLT2i and OAM episodes in our study might be attributed to the cardioprotective potential of certain OAM other than SGLT2i, such as metformin and GLP1a. For example, Onoue et al. (*N* = 315, 2008–2021) found that metformin was associated with a lower incidence of symptomatic HF within one year of initiating anthracyclines (HR = 0.35, 95% CI = 0.14–0.90; *p* = 0.03).^39^ The mechanisms of metformin, including anti-inflammatory effects and reactive oxygen species inhibition, may prevent anthracycline-induced CM.^40^ GLP1a has also been shown to reduce the risk of major adverse cardiovascular outcomes, though they are less effective at preventing HF compared to SGLT2i.^41^ Our study included 14.3% metformin and 14.7% GLP1a users in the OAM group, which may potentially mask significant associations between SGLT2i use and reduced cardiovascular outcomes.

The limited sample size of the SGLT2i group and the impact of non-CV mortality may have also contributed to the insufficient statistical power. Although our results did not reach statistical significance, the CIs for several outcomes do not preclude the possibility of a clinically meaningful benefit with SGLT2i use. In addition, all-cause death was treated as a competing risk, and we could not differentiate between cardiovascular and non-cardiovascular mortality. A potential reduction in non-CV deaths with SGLT2i use may attenuate the potential cardiovascular benefits.^42^

Consistently lower point estimates across all subgroups may suggest potential cardioprotective effects of SGLT2i in older women with breast cancer following anthracycline or trastuzumab treatment, though most findings should be interpreted cautiously due to limited sample sizes. Reductions in cardiovascular composite endpoint and incident HF/CM with individual SGLT2i indicate a potential class effect.^43^ However, RCTs on SGLT2i’s efficacy in mitigating cardiotoxicity among patients with cancers are limited, with only the EMPACT (Empagliflozin in the Prevention of Cardiotoxicity in Cancer Patients Undergoing Chemotherapy Based on Anthracyclines, NCT05271162) trial focusing on empagliflozin.

The cardioprotective effects of SGLT2i appeared to be more pronounced, though not statistically significant, in women receiving anthracyclines compared to those treated with trastuzumab. The different cardiotoxic profiles of anthracyclines and trastuzumab might explain these findings in our study. Anthracyclines are associated with type 1 cardiotoxicity, causing irreversible damage to cardiomyocytes.^44^ The irreversible nature of this damage might contribute to the more pronounced cardioprotective effect of SGLT2i in patients treated with anthracyclines in our study, as these medications may mitigate the continuous and accumulating cardiotoxic burden. In contrast, trastuzumab is associated with type 2 cardiotoxicity characterized by dose-independent, potentially reversible myocardial damage.^45^ The potential for myocardial recovery after discontinuation of trastuzumab could reduce the relative impact and observable benefits of SGLT2i during the follow-up period, possibly explaining the less evident cardioprotective benefits of SGLT2i in this subgroup. Thus, the cardioprotective strategies should also be evaluated based on the specific cardiotoxic cancer treatment regimen they receive.

From a clinical perspective, the consistent direction of effect estimates may suggest a potential signal of benefit, particularly among patients exposed to anthracyclines. Given the well-established cardioprotective effects of SGLT2i in other high-risk populations with T2DM, their use may be reasonable to consider in older breast cancer survivors with elevated baseline cardiovascular risk, especially when glycemic control is also a therapeutic priority. Due to the exploratory nature of the subgroup analyses, the inclusion of active comparators with known or suspected cardioprotective properties, and the limited sample size, these findings should not be interpreted as sufficient evidence to guide routine clinical prioritization of SGLT2is for cardioprotection in this setting. Instead, the results highlight the need for further prospective investigation.

### Limitations

This study has several limitations. First, while we employed sophisticated epidemiological designs and statistical methods with competing risk, potential bias from unmeasured confounders in administrative data cannot be completely eliminated. However, robust propensity matching with comprehensive covariates was employed to minimize confounding effects. Second, we could not account for LVEF, diastolic function, or cardiac biomarkers such as troponin and natriuretic peptides, which hinder the identification of asymptomatic CTRCD, may have led to under-ascertainment of secondary outcomes such as incident HF or CM. In addition, the SEER-Medicare database did not adequately capture the cumulative dosage and treatment duration of anthracyclines, though this limitation equally affects both SGLT2i and OAM groups. Third, information on treatment discontinuation due to cardiotoxicity was not available. This might limit our ability to evaluate the full spectrum of cardiotoxic effects and their impact on treatment decisions. Fourth, our cohort was limited to women aged 65.5 years and older with early-EBC. Differences in comorbid burden, drug metabolism, and baseline cardiovascular risk between older and younger adults may influence both the incidence of cardiotoxicity and the cardioprotective effects of SGLT2i, which may limit the generalizability of this study to younger cancer survivors or other types of cancers. Fifth, due to the episode-based design, a single patient could contribute episodes to both the SGLT2i and OAM groups. Although a robust variance estimate was used to adjust for this, results may still trend toward the null. Last but not least, a limited follow-up period and small sample size may have reduced statistical power despite large effect sizes. Nonetheless, consistent results favoring SGLT2i over OAM in primary and subgroup analyses provide valuable real-world insights into their use in older women treated with anthracyclines or trastuzumab.

## Conclusion

Our study revealed a potential reduced risk of CV events among older women with EBC treated with SGLT2i compared to those treated with OAM following therapies with anthracycline or trastuzumab. While these results did not reach statistical significance, which may be attributed to the limited sample size, short follow-up period, and inclusion of active comparators, they suggest a protective trend of SGLT2i against CV events in the older breast cancer population. While the consistent observation of lower point estimates for both composite CV outcomes and incident HF/CM across various subgroups may suggest intriguing cardioprotective potential benefits of SGLT2i, further investigation through RCTs is necessary to confirm any cardioprotective effect of SGLT2i in patients with cancer.

## Supplemental Material

sj-docx-1-tam-10.1177_17588359251378245 – Supplemental material for Cardioprotective effectiveness of SGLT2 inhibitors in older diabetic women with early-stage breast cancer following anthracycline- and/or trastuzumab-based treatment Supplemental material, sj-docx-1-tam-10.1177_17588359251378245 for Cardioprotective effectiveness of SGLT2 inhibitors in older diabetic women with early-stage breast cancer following anthracycline- and/or trastuzumab-based treatment by Yi-Shao Liu, Jamie C. Barner, Kenneth A. Lawson, Yan Liu and Chanhyun Park in Therapeutic Advances in Medical Oncology

## Declarations

**Ethics approval and consent to participate:** This study was reviewed and approved by the Institutional Review Board (IRB) of The University of Texas at Austin (IRB Number: STUDY00002248). The IRB determined that this protocol meets the criteria for exemption from IRB review under 45 CFR 46.104 (4) Secondary research on data or specimens (no consent required).

**Consent for publication:** Not applicable.

**Availability of data and materials:** Researchers can submit an application to access the new data linkage through the SEER-Medicare data request process, available at https://healthcaredelivery.cancer.gov/seermedicare/obtain/requests.html
