# Supplementary material for: Cardioprotective effectiveness of SGLT2 inhibitors in older diabetic women with early-stage breast cancer following anthracycline- and/or trastuzumab-based treatment
Source: Ther Adv Med Oncol. 2025 Sep 23;17:17588359251378245. doi: 10.1177/17588359251378245 (PMC12461033; doi:10.1177/17588359251378245)
Supplement: sj-docx-1-tam-10.1177_17588359251378245 – Supplemental material for Cardioprotective effectiveness of SGLT2 inhibitors in older diabetic women with early-stage breast cancer following anthracycline- and/or trastuzumab-based treatment [file sj-docx-1-tam-10.1177_17588359251378245.docx]

**Supplementary Materials**

Supplementary Table 1. International Classification of Diseases, Ninth (ICD-9) and Tenth Revision (ICD 10) Diagnosis Codes for Outcomes and Baseline Comorbidities 2

Supplementary Table 2. Covariates for the Non-parsimonious Propensity Score Estimates 3

Supplementary Table 3. Event Counts, Crude Incidence Rates, and Hazard Ratios for Episodes Initiated with SGLT2i or OAM in the Subgroup Analysis of Individual SGLT2i 4

Supplementary Table 4. Event Counts, Crude Incidence Rates, and Hazard Ratios for Episodes Initiated with SGLT2i or OAM in the Subgroup Analysis Based on Previous CVDs and Cardiotoxic Cancer Treatment 5

Supplementary Table 5. STROBE Statement—Checklist of items that should be included in reports of cohort studies 6

**Supplementary Table 1**. **International Classification of Diseases, Ninth (ICD-9) and Tenth Revision (ICD-10) Diagnosis Codes for Outcomes and Baseline Comorbidities.**

| **Disease** | **ICD-9 diagnosis codes^a^** | **ICD-10 diagnosis codes^a^** |
| --- | --- | --- |
| **Cardiovascular diseases** | | |
| Heart failure | 428 | I50 |
| Cardiomyopathy | 425 | I42, I43 |
| Stroke | 430, 431, 433.x1, 434.x1, 435, 436, 362.3 | I60, I61, I63, I64, H34.1, G45 |
| Myocardial infarction | 410, 412 | I21, I22, I25.2 |
| Angina | 411, 413, 414.0 | I20.0 |
| Peripheral vascular disease | 093.0, 440, 441, 443.1–443.9, 447.1, 557.1, 557.9, V43.4 | I70, I71, I73.1, I73.8, I73.9, I77.1, I79.0, I79.2, K55.1, K55.8, K55.9, Z95.8, Z95.9 |
| Atrial fibrillation | 427.3 | I48 |
| Valvular heart diseases | 394.0, 394.1, 394.2, 395.0, 395.1, 395.2, 397.0, 424.0, 424.1, 424.2 | I05.0, I05.1, I05.2, I06.0, I06.1, I06.2, I07.0, I07.1, I07.2, I34.0, I34.1, I34.2, I35.0, I35.1, I35.2, I36.1, I36.2 |
| Arrhythmia | 426.0, 426.13, 426.7, 426.9, 426.10, 426.12, 427.0–427.4, 427.6–427.9, 785.0, 996.01, 996.04, V45.0, V53.3 | I44.1–I44.3, I45.6, I45.9, I47–I49.x, R00.0, R00.1, R00.8, T82.1, Z45, Z95.0 |
| Hyperlipidemia | 272.0, 272.1, 272.2, 272.3, 272.4 | E78 |
| **Other diseases** | | |
| Renal disease | 403.01, 403.11, 403.91, 404.02, 404.03, 404.12, 404.13, 404.92, 404.93, 582, 583.0–583.7, 585, 586, 588.0, V42.0, V45.1, V56 | I12.0, I13.1, N03.2–N03.7, N05.2–N05.7, N18, N19, N25.0, Z49.0–Z49.2, Z94.0, Z99.2 |
| Severe hypoglycemia | 251, 962.3 | E10.0, E11.0, E12.0, E13.0, E14.0, E11.6A, E16.0–E16.2 |
| Chronic obstructive pulmonary disease | 416.8, 416.9, 490–505, 506.4, 508.1, 508.8 | I27.8, I27.9, J40–J47, J60–J67, J68.4, J70.1, J70.3 |
| Obesity | 278 | E66 |
| Type 1 diabetes | 250.x1, 250.x3 | E10 |
| Diabetes | 250.xx | E11 |

ICD, International Classification of Diseases.

^a^ For some diseases, ICD-9 codes were converted from ICD-10 codes based on the 2017 General Equivalence Mappings files.

**Supplementary Table 2. Covariates for the Non-parsimonious Propensity Score Estimates.**

| **Category** | **Covariates** |
| --- | --- |
| Sociodemographic characteristics | Age on the index date, race/ethnicity, region of residence, metro/non-metro residence, marital status, poverty level |
| Presence of baseline comorbidities | Heart failure, cardiomyopathy, valvular heart diseases, myocardial infarction, angina, arrhythmia, stroke, peripheral vascular disease, hyperlipidemia, chronic kidney disease, severe hypoglycemia, chronic obstructive pulmonary disease, obesity |
| Baseline medications | Cardiovascular-related medication:  Dexrazoxane, aspirin, beta-blockers, angiotensin-converting enzyme inhibitors (ACEIs), angiotensin receptor blockers (ARBs), statins, calcium-channel blockers, diuretics, direct oral anticoagulants (DOACs), P2Y12 inhibitors, warfarin, other antiplatelets  Anti-diabetic medications:  Insulins, metformin, sulfonylureas, metiglinide analogues, acarbose, thiazolidinediones, dipeptidyl-peptidase 4 inhibitors (DPP4i), glucagon-like peptide-1 agonists (GLP-1a) |
| Breast cancer-related factors | SEER summary stage, histology grade, tumor size, breast cancer subtype, surgery, radiation, cardiotoxic cancer treatment exposure (anthracycline or trastuzumab) |
| Others | Index year, diagnosis year, time gap between the index date and cardiotoxic cancer treatment date |

SEER, Surveillance, Epidemiology, and End Results.

**Supplementary Table 3.** **Event Counts, Crude Incidence Rates, and Hazard Ratios for Episodes Initiated with SGLT2i or OAM in the Subgroup Analysis of Individual SGLT2i.**

| Outcomes | Canagliflozin subgroup | | Dapagliflozin subgroup | | Empagliflozin subgroup | |
| --- | --- | --- | --- | --- | --- | --- |
|  | OAMs | Canagliflozin | OAMs | Dapagliflozin | OAMs | Empagliflozin |
| **N** | 192 | 51 | 93 | 26 | 196 | 52 |
| **Composite endpoint (HF, stroke, MI, and arrhythmia)** | | | | | | |
| Event counts^b^ (crude incidence rate^a^) | 36 (8.8) | <11 (7.3) | 16 (11.0) | <11 (5.0) | 19 (8.3) | <11 (5.6) |
| csHR (95% CI) | 1.0 (ref) | 0.83 (0.45 to 1.56) | 1.0 (ref) | 0.46 (0.12 to 1.72) | 1.0 (ref) | 0.67 (0.22 to 2.01) |
| **HHF** | | | | | | |
| Event counts^b^ (crude incidence rate^a^) | <11 (0.6) | <11 (0.7) | <11 (0.6) | 0 (0.0) | 0 (0.0) | 0 (0.0) |
| csHR (95% CI) | 1.0 (ref) | 1.17 (0.41 to 3.33) | 1.0 (ref) | 0 | 1.0 (ref) | 1.0 |
| **Incident HF or CM** | | | | | | |
| Event counts (crude incidence rate^a^) | 27 (6.4) | <11 (3.9) | 13 (8.8) | <11 (5.0) | 19 (8.3) | <11 (7.6) |
| csHR (95% CI) | 1.0 (ref) | 0.63 (0.45 to 1.56) | 1.0 (ref) | 0.57 (0.16 to 2.07) | 1.0 (ref) | 0.93 (0.34 to 2.52) |

CI, confidence interval; CM, cardiomyopathy; csHR, cause-specific hazard ratio; HHF, hospitalization due to heart failure; HF, heart failure; MI, myocardial infarction; OAM, other anti-diabetic medications; SGLT2i, sodium-glucose cotransporter-2 inhibitors.

^a^ Per 100 person-years.
^b^ Numbers less than 11 are suppressed per SEER-Medicare data use agreement.

**Supplementary Table 4. Event Counts, Crude Incidence Rates, and Hazard Ratios for Episodes Initiated with SGLT2i or OAM in the Subgroup Analysis Based on Previous CVDs and Cardiotoxic Cancer Treatment.**

| Outcomes | **With previous CVDs** | | **Without previous CVDs** | |
| --- | --- | --- | --- | --- |
|  | OAMs | SGLT2is | OAMs | SGLT2is |
| N | 141 | 42 | 286 | 80 |
| **Composite endpoint (HF, stroke, MI, and arrhythmia)** | | | | |
| Event counts^b^ (crude incidence rate^a^) | 29 (14.0) | <11 (12.5) | 31 (6.1) | <11 (4.7) |
| csHR (95% CI) | 1.0 (ref) | 0.85 (0.41 to 1.77) | 1.0 (ref) | 0.78 (0.41 to 1.51) |
| **HHF** | | | | |
| Event counts^b^ (crude incidence rate^a^) | <11 (1.3) | 0 (0.0) | 0 (0.0) | 0 (0.0) |
| csHR (95% CI) | 1.0 (ref) | 0 | 1.0 (ref) | 1 |
| **Incident HF or CM** | | | | |
| Event counts^b^ (crude incidence rate^a^) | 21 (9.8) | <11 (6.8) | 23 (4.5) | <11 (4.7) |
| csHR (95% CI) | 1.0 (ref) | 0.66 (0.24 to 1.82) | 1.0 (ref) | 1.12 (0.54 to 2.31) |
| Outcomes | **Anthracycline-only** | | **Trastuzumab** | |
|  | OAMs | SGLT2is | OAMs | SGLT2is |
| N | 167 | 47 | 271 | 76 |
| **Composite endpoint (HF, stroke, MI, and arrhythmia)** | | | | |
| Event counts^b^ (crude incidence rate^a^) | 24 (8.7) | <11 (3.5) | 39 (9.4) | <11 (8.7) |
| csHR (95% CI) | 1.0 (ref) | 0.41 (0.13 to 1.32) | 1.0 (ref) | 0.94 (0.53 to 1.69) |
| **HHF** | | | | |
| Event counts^b^ (crude incidence rate^a^) | <11 (0.3) | 0 (0.0) | <11 (0.4) | <11 (0.8) |
| csHR (95% CI) | 1.0 (ref) | 0 | 1.0 (ref) | 1.92 (1.46 to 2.52) |
| **Incident HF or CM** | | | | |
| Event counts^b^ (crude incidence rate^a^) | 16 (5.6) | <11 (3.5) | 32 (7.6) | <11 (6.8) |
| csHR (95% CI) | 1.0 (ref) | 0.64 (0.21 to 1.92) | 1.0 (ref) | 0.95 (0.46 to 1.94) |

CI, confidence interval; CM, cardiomyopathy; CVDs, cardiovascular diseases; csHR, cause-specific hazard ratio; HHF, hospitalization due to heart failure; HF, heart failure; MI, myocardial infarction; OAM, other anti-diabetic medications; SGLT2i, sodium-glucose cotransporter-2 inhibitors.

^a^ Per 100 person-years.
^b^ Numbers less than 11 are suppressed per SEER-Medicare data use agreement.

**Supplementary Table 5.** STROBE Statement—Checklist of items that should be included in reports of cohort studies

|  | | Item No | Recommendation | Page No |
| --- | --- | --- | --- | --- |
| **Title and abstract** | | 1 | (*a*) Indicate the study’s design with a commonly used term in the title or the abstract | 1, 2 |
|  |  |  | (*b*) Provide in the abstract an informative and balanced summary of what was done and what was found | 1, 2 |
| Introduction | | | | |
| Background/rationale | | 2 | Explain the scientific background and rationale for the investigation being reported | 4 |
| Objectives | | 3 | State specific objectives, including any prespecified hypotheses | 4 |
| Methods | | | | |
| Study design | | 4 | Present key elements of study design early in the paper | 5, 6 |
| Setting | | 5 | Describe the setting, locations, and relevant dates, including periods of recruitment, exposure, follow-up, and data collection | 5, 6 |
| Participants | | 6 | (*a*) Give the eligibility criteria, and the sources and methods of selection of participants. Describe methods of follow-up | 6, 7 |
|  |  |  | (*b*) For matched studies, give matching criteria and number of exposed and unexposed | 7, 8 |
| Variables | | 7 | Clearly define all outcomes, exposures, predictors, potential confounders, and effect modifiers. Give diagnostic criteria, if applicable | 6, 7 |
| Data sources/ measurement | | 8* | For each variable of interest, give sources of data and details of methods of assessment (measurement). Describe comparability of assessment methods if there is more than one group | 5 |
| Bias | | 9 | Describe any efforts to address potential sources of bias | 6-8 |
| Study size | | 10 | Explain how the study size was arrived at | na |
| Quantitative variables | | 11 | Explain how quantitative variables were handled in the analyses. If applicable, describe which groupings were chosen and why | 5, 6 |
| Statistical methods | | 12 | (*a*) Describe all statistical methods, including those used to control for confounding | 7, 8 |
|  |  |  | (*b*) Describe any methods used to examine subgroups and interactions | 8 |
|  |  |  | (*c*) Explain how missing data were addressed | na |
|  |  |  | (*d*) If applicable, explain how loss to follow-up was addressed | na |
|  |  |  | (*e*) Describe any sensitivity analyses | 8 |
| Results | | | |  |
| Participants | | 13* | (a) Report numbers of individuals at each stage of study—eg numbers potentially eligible, examined for eligibility, confirmed eligible, included in the study, completing follow-up, and analysed | 9 |
|  |  |  | (b) Give reasons for non-participation at each stage | 9 |
|  |  |  | (c) Consider use of a flow diagram | Figure 2 |
| Descriptive data | | 14* | (a) Give characteristics of study participants (eg demographic, clinical, social) and information on exposures and potential confounders | 9 |
|  |  |  | (b) Indicate number of participants with missing data for each variable of interest | Table 1 |
|  |  |  | (c) Summarise follow-up time (eg, average and total amount) | 9, 10 |
| Outcome data | | 15* | Report numbers of outcome events or summary measures over time | 9, 10 |
| Main results | 16 | (*a*) Give unadjusted estimates and, if applicable, confounder-adjusted estimates and their precision (eg, 95% confidence interval). Make clear which confounders were adjusted for and why they were included | | Table 2 |
|  |  | (*b*) Report category boundaries when continuous variables were categorized | | na |
|  |  | (*c*) If relevant, consider translating estimates of relative risk into absolute risk for a meaningful time period | | Table 2 |
| Other analyses | 17 | Report other analyses done—eg analyses of subgroups and interactions, and sensitivity analyses | | 10, 11 |
| Discussion | | | | |
| Key results | 18 | Summarise key results with reference to study objectives | | 12 |
| Limitations | 19 | Discuss limitations of the study, taking into account sources of potential bias or imprecision. Discuss both direction and magnitude of any potential bias | | 15 |
| Interpretation | 20 | Give a cautious overall interpretation of results considering objectives, limitations, multiplicity of analyses, results from similar studies, and other relevant evidence | | 12, 13 |
| Generalisability | 21 | Discuss the generalisability (external validity) of the study results | | 15 |
| Other information | | | | |
| Funding | 22 | Give the source of funding and the role of the funders for the present study and, if applicable, for the original study on which the present article is based | | 17 |

*Give information separately for exposed and unexposed groups.

**Note:** An Explanation and Elaboration article discusses each checklist item and gives methodological background and published examples of transparent reporting. The STROBE checklist is best used in conjunction with this article (freely available on the Web sites of PLoS Medicine at http://www.plosmedicine.org/, Annals of Internal Medicine at http://www.annals.org/, and Epidemiology at http://www.epidem.com/). Information on the STROBE Initiative is available at http://www.strobe-statement.org.
